# Supplementary material for: Elastic energy savings and active energy cost in a simple model of running
Source: PLoS Comput Biol. 2021 Nov 23;17(11):e1009608. doi: 10.1371/journal.pcbi.1009608 (PMC8651147; doi:10.1371/journal.pcbi.1009608)
Supplement: Attachment — Submitted filename: R1_ResponseToReviewerComments.docx [file pcbi.1009608.s002.docx]

*We appreciate the reviewers’ thoughtful comments and thank them for their careful reading of the manuscript. We believe we have addressed any previous concerns. Please note, blue text indicates our response to comments, while red text indicates excerpts of revisions made to the manuscript. Line numbers are referenced to the version of the manuscript with tracked changes.*

# Reviewer #1:

**Summary:**

The authors offer an improved computational model of the energetic cost of running in humans by accounting for aspects of running that are generally ignored in models of legged locomotion. These aspects include losses in the virtual leg springs, losses due to collision with the ground, the rate at which the leg musculature/actuator produces work, and the cost of negative work. It is common sense that the two losses might be important and the authors cite sufficient literature to argue that the rate of work is important. By taking these additional costs into consideration, the authors show a cost breakdown that corresponds roughly with predictions of the percent of energetic cost from active muscle work from the literature (assuming reasonable elastic energy return from the tendons). They are also able to extend their model to capture the changes to energetic cost of a human running up and down slopes. Overall, this paper is a nice contribution to the literature on dynamic legged locomotion.


**Major comments:**

I am concerned that the flat ground human running data all comes from one individual. The paper that this single individual's data was drawn from had 10 young male runners -- is there a reason that only one runner's data was selected for comparison to the model?

We show representative data as an example of basic running mechanics already described extensively in the literature. We only perform descriptive, qualitative comparisons of running mechanics, with no statistical claims. To make it clearer that the data show nothing new, we have added more citations of papers showing similar features:

Lines 398-406: “The model also reproduces some features that the classical Spring-mass model cannot, such as the brief initial peak at touchdown (from collision, 31, Fig. 7b) and a more gradual decrease in force than the increase (temporal asymmetry, 30, Fig. 7b). The model collision produces a transient burst of negative power (46) followed by elastic energy storage and return (about 53%; Fig. 7c), as well as slightly non-linear vertical work loop curves (as in 4, Fig. 7d), qualitatively similar to human.”

Note that when we used human data more quantitatively (e.g., Fig. 8a), we used (previously published) empirical metabolic data from multiple subjects (N=10).

In this paper, the spring-actuator model has the spring (with damping) on top next to the center of mass and the actuator lower down in the leg. Intuitively, I expect to see the actuator closer to the center of mass and the damped spring closer to the toe. If the actuator is a rough analog for the muscles of the leg and the damped spring is a rough analog for the tendons, then actuator-on-top seems to be the more common body plan for mammals and birds. Also, having the damped spring being close to the toe would allow it to contribute to rejecting perturbations from the ground and take up energy during collisions -- legged robots with series elastic actuators tend to put the springs nearer to the end effectors for this reason. A sentence or two explaining why the authors made this modeling choice would be very helpful to me as a reader.

We have adjusted the ordering of spring and actuator as the reviewer prefers (see Fig. 2, 4, 6-9). These are lumped parameter elements whose ordering makes no difference to the model. However, we agree that a distal spring-damper might be considered more biological.

Finally, I want to point out an option which is available to the authors. Since they have a comparison between the actuator-only and spring-actuator models, they could comment on the differences between biological and robotic legged locomotors. Actuator-only locomotion schemes are common for robots with legs, and some discussion of when actuator-only legged robots can be used to test models of locomotion in the physical world would increase the audience for the paper. There are also bipedal robots like Cassie which have springs in their lower legs (whereas the model in this paper puts the springs closer to the center of mass), and the authors could comment on the choice of spring placement. I understand that relating the results to legged robots is outside the current scope of the paper and the authors should not interpret this suggestion as a requirement -- merely a suggestion if they are interested in broadening their audience. Anecdotally, I have found that researchers working with legged robots have a lot of interest in how to judiciously use damping to improve locomotion performance.

We agree and have added a discussion paragraph regarding robotics to the Discussion section:

Lines 608-623: “**Implications for legged robots**

“The present study may be relevant to running robots that employ spring-like behavior. In the SLIP (spring-loaded inverted pendulum) paradigm, a controller causes the overall leg to behave like a spring, despite internal dissipation. Robotic dissipation includes actuator heat and transmission losses (analogous to actuator work efficiency and hysteresis in our model) as well as from interactions with the environment (e.g. collisions) (58). With SLIP control, the stiffness is sometimes selected to resemble animal gait (59), just as our model resembles human. Our results suggest that SLIP may actually be reasonably economical, because our model, similar to others modeling dissipation with more detail (27,28), yield optimal force profiles that are still remarkably spring-like. However, closer examination also reveals that better economy is achieved with small but significant differences such as force asymmetry (Fig. 7). We also note that matching a gait to human or animal is not necessarily the best option for economy, which might favor a quite different stiffness (Fig. 8b). There are also potential benefits to including passive dissipation, which can yield improved stability (29) and velocity estimation, which is considered important for robust control (60,61). Robotic controllers can take advantage of passive dissipation by modeling it explicitly (e.g., 62) and will respond differently when optimized for economy.”

**Minor comments:**

Why is "Spring-mass model" capitalized in this way? I expect either "spring-mass model" or, if the authors want to refer to the model using an acronym, "Spring-Mass Model (SMM)". Similarly, I suggest changing "Actuator-only" to "actuator-only" and "Actuated Spring-mass" to "actuated spring-mass".

We appreciate the reviewer’s suggestion, but prefer to capitalize the names of the models as proper nouns, to make them more distinct from more generic usage of “mass” and “spring” not in the context of the model, for example when describing the human body or tendons. We also prefer to avoid use of acronyms.

29: Even if the tendons are very good springs, it would be impossible for them to be totally lossless. We would expect them to dissipate SOME energy, requiring the muscles to add that energy back in order for the animal to maintain a steady-state run. I would suggest taking out the phrase "and why the muscles do any work at all". If the authors wish to make the point that their model accounts for dissipation in the tendons where other models do not, I think it is important that they clarify this. Before reading the paper, it was not obvious to me whether the classical model the authors refer to assumes lossless springs or not.

Indeed, the classical model does use a lossless spring, so we have adjusted the explanation to make this clearer. In Introduction, Lines 40-42: “…the limb acts elastically to support and redirect the body center of mass (CoM) between flight phases and all mechanical energy is conserved throughout each step.”

Line 64: “Human stance phases resemble motion of a spring-mass system with no energy loss…”

Author Summary: “… it does not explain why running still requires considerable effort, and why the muscles still do substantial work.”

61: Flight --> flight

We did not make this change, because “Flight” is capitalized as labelled in the figure.

Since the authors make the point that models of legged locomotion rarely consider damping, it seems important to include some other recent work on the importance of including damping in computational models. I am personally interested in how the authors would relate their work to this article which came out in August 2020, but the authors may find papers that are a better fit (I realize that this paper is talking about economy of motion rather than stability):
Heim S, Millard M, Le Mouel C, Badri-Spröwitz A. 2020 A little damping goes a long way: a simulation study of how damping influences task-level stability in running. Biol. Lett. 16: 20200467. <http://dx.doi.org/10.1098/rsbl.2020.0467>

We have added more background about similar models with parallel damping, e.g. lines 101-104: “For organisms, similar models have been used to explore stability (29) and economical strategies (30) of terrain navigation. Still, while dissipation has been characterized empirically (e.g., 31), most running energetics models have not included such dissipation with series elasticity and actuation to explain energy cost.”

We have also added some discussion regarding the utility of damping in robotics, including stability. Lines 619-623: “There are also potential benefits to including passive dissipation, which can yield improved stability (29) and velocity estimation, which is considered important for robust control (60,61). Robotic controllers can take advantage of passive dissipation by modeling it explicitly…”

# Reviewer #2:

The manuscript “Elastic energy savings and active energy cost in a simple 2 model of running” reports on the results of a simulation/optimization study on a conceptual running model that investigates different potential contributions to energy expenditure. The model is a simple point mass with a mass-less leg that features a compliant element with parallel damping and series actuation. Energy optimal motions are found through numerical optimization for which the cost function is composed of a work term (integral of weighted positive and negative mechanical power) and a force-rate term (integral of force-rate squared). The model is most similar to the one presented in [1] for which energy-optimal motions have also been identified. The main difference with regard to the model is the inclusion of (virtual) collision losses. I would consider the main contribution of the paper to be the proposed consideration of a force-rate cost term, which is carefully studied. In particular, this force-rate term might stipulate a pseudo-elastic behavior (with active negative work) that could explain the apparent elastic characteristics of running while also explaining the associated energy cost. Without being able to provide conclusive evidence that this is indeed what happens in biological systems, the results suggest that some type of force-rate term (not necessarily exactly as the one presented) might explain characteristics observed in human running and they clearly establish a sound alternative explanation to a purely elastic behavior. These conclusion are formulated with the appropriate care, and limitations of the used model and methods are adequately addressed.

Overall, the paper is very well written and nicely framed. My comments are mostly positive, yet I still see some potential for improvements. Most importantly the inclusion of a discussion of currently unreported prior work. With regard to the presentation, the explanations in the manuscript sometimes require the reader to infer some intermediate steps themselves. This could be clarified to help with the readability of the paper.

• While the paper is well grounded in the existing literature, it seems to be missing some recent work out of the collaborations of Monika Daley and Jonathan Hurst (e.g., [1]), which studies work-minimal motions of running birds using a nearly identical model with damping losses and active (series elastic) inputs. I feel that these contributions need to be discussed in the context of your proposed model (L101).

We have added this reference to the introduction and adjusted the wording to clarify our contribution. Lines 101-104: “For organisms, similar models have been used to explore stability (29) and economical strategies (30) of terrain navigation. Still, while dissipation has been characterized empirically (e.g., 31), most running energetics models have not included such dissipation with series elasticity and actuation to explain energy cost.”

• With simple models as the one presented in this paper, a particular important question is the choice of model parameters. These are well justified in the manuscript, but I would suggest to summarize them in a table for a better overview.

As suggested, we have added Table 1 at Line 208, which summarizes parameter values (in Methods).

• What puzzled me initially was the different physical nature of the actuators in Fig 2. As the actuator in 2b acts against a mass M, I immediately envisioned it as a force-source, whereas the actuator in 2c appears to be a kinematic constraint (which is not acting against a mass). Furthermore, it was not immediately clear how the force-rate would be computed for case 2b. These apparent differences are cleverly resolved by using the jerk of the leg length as input, yet that information is hidden in the supplementary information. I would suggest to expand a little bit on this issue after line 181 and explicitly state that jerk is the input to all actuators (and force-rate a dependent quantity that follows from this kinematic constraint).

The reviewer is correct. We have therefore adjusted the Methods to explain that leg jerk is the control variable for our model, in part to facilitate calculations of force rate and resulting cost. Lines 161-164: “The main control variable was the time-varying actuator length during the leg’s stance (specifically, its third derivative ${\overset{⃛}{L}}_{m}(t)$ was used to allow for calculations of force rate during implementation)…”

We have also added some high-level clarification to explain how force rate is calculated and point readers to the Appendix for more explicit detail. Lines 181-183: “…*Ḟ_m_* is the first derivative of actuator force and *ε* is a cost coefficient that converts the mechanical quantity into units of energy. Actuator force and force rate were determined from the spring-damper force and its derivative, respectively (see S1 Appendix in the Supporting Information for details).”

• While the paper is very mathematical in its methods, the description of the different scenarios relies often purely on verbal descriptions. It might help for a more mathematically focused audience to include the relevant symbols whenever possible. (e.g., L179: “Model states included the position (x,y) and velocity (\dot{x}, \dot{y}) of the point mass body and the first and second derivatives of the actuator’s length (\dot{l} and \ddot{l}) to …”; L192: “tendon stiffness k … and force-rate coefficient \epsilon” and in many other places.)

We have added symbols next to word descriptions as suggested.

• L154: a) Shouldn’t this read “L_m(t)”? b) Wouldn’t it be more precise to state that the jerk of L_m(t) was the control input. I know that technically defining L_m(t) defines the jerk, but there are some implicit assumptions in play that, for example, require L_m(t) to be three times differentiable for a trajectory to be physically realizable and to have a well-defined finite work-rate cost.

We changed the symbol to L_m(t). Also, we have clarified that the third derivative was used as the control variable during implementation to facilitate calculation of force rate and associated cost. Lines 161-163: “The main control variable was the time-varying actuator length during the leg’s stance (specifically, its third derivative ${\overset{⃛}{L}}_{m}(t)$ was used to allow for calculations of force rate during implementation)…”

• Eq 3 uses the lunate epsilon symbol, whereas elsewhere in the document the Greek lowercase epsilon is used.

We have made epsilons consistent.

• L172/L193/L246/L275: From a philosophical point of view, the nature of \epsilon could be two-fold. One can either think of it as a relative weight in a weighted cost function (in which case it would be placed better in eq. 1 and not in eq.3) or as a biomechanical parameter that has a clearly defined unit and needs to be identified (and for which we don’t know a-priori if it is unequal to 0). I believe the paper only wants to do the latter, yet sometimes falls into wording that would be more appropriate for the former (such as: “With work as the sole cost”). I would a) suggest to be a bit more precise in the wording (“for a cost coefficient \epsilon=0”) b) to define the range to be from 0 to 0.02) , and c) to immediately clarify the units and nature of \epsilon.

We do indeed mostly consider the force-rate cost coefficient as a biomechanical parameter that converts the mechanical quantity (integral of force rate squared) into non-dimensional units of energy. We have adjusted wording throughout the paper to match this sentiment, as suggested. We also updated the range of cost coefficients to include zero value and added some clarification when the parameter is introduced. Lines 181-182: “…*ε* is a cost coefficient that converts the mechanical quantity into units of energy.”

• Along those same lines, I would suggest to generally avoid expressions such as “with *work* as the sole cost,…”, as it lacks a bit precision. I was first thinking of a completely different cost function of mechanical work or positive mechanical work, before realizing that you meant E_W, which is not work, but the “cost of work”. (I know this is getting very detailed, but the paper is at a point where only very detailed comments will make it even better).

We have adjusted the wording for clarity in a number of lines and figures (e.g., Lines 230, 288, 297, 317, 573), to indicate when the force-rate coefficient is zero.

• L328: To clarify it might help to state that the work is needed to compress the spring to increase the force. I was first puzzled since force does not show up in any cost term.

We agree this sentence is confusing and have removed it.

• L365: Your reporting of the efficiency constants here reminded me that these are not reported in the first part of the results. Their particular choice will likely not influence the results as long as negative work cannot be recovered, but their choice influences the relative weight of \epsilon, so they must be reported. Again, a table (maybe in combination with the one requested above) would help with clarity.

We have added a table that reports parameter values (including work efficiency values) used in optimizations (see Table 1 in Methods).

• L364: how was \epsilon and k determined? Are these parameters fitted to data or follow from optimality criteria? This seems very important, as a key message of the following results is similarity to human data.

These parameters were adjusted by hand until the model outputs approximately matched human data. We have adjusted the wording to hopefully improve clarity. Lines 391-392: “A single force-rate coefficient is selected (*ε** of 0.5 · 10^-3^) to approximately match the model’s outputs to human data, along with stiffness…”

And also added explanation to the Methods section. Lines 205-207: “Furthermore, a single set of nominal parameter values (force-rate coefficient *ε*, spring stiffness *k* and negative and positive work efficiency *η^-^* and *η^+^*) were selected for comparisons with human ground reaction forces and metabolic data (see Table 1)”

• L373/L508: If I remember correctly, this shape is also reported in the above mentioned reference.

We have cited this work as suggested. Lines 555-558: “Other models have also demonstrated the economy of asymmetrical trajectories in bird running (30) but do not include a brief transient in ground reaction force and work at touchdown (Fig. 7b and c), observed prominently in human foot contact.”

[1] “Don’t break a leg: running birds from quail to ostrich prioritise leg safety and economy on uneven terrain”, The Journal of Experimental Biology (2014) 217, 3786-3796
